# Lower respiratory infections (LRIs) etiologies in hospitalized children in New Caledonia: a PERCH pilot study

**Published:** 2011-01-10

**Authors:** V Zurawski, A Driscoll, A Deluca, M Knoll, D Murdoch, O O’Connor, M Dupont-Rouzeyrol, I Missotte, J Moïsi, L Besson-Leaud, C Chevalier, V Debarnot, O Levine, S Mermond

**Affiliations:** 1 Pasteur Institute of New Caledonia, Noumea, New Caledonia; 2 The Johns Hopkins Bloomberg School of Public Health, Baltimore, Maryland, USA; 3 Department of Microbiology, Canterbury Health Laboratories, Christchurch, New Zealand; 4 Paediatric Ward, Territorial Hospital Center of Magenta, Noumea, New Caledonia; 5 Paediatric Emergency Unit, Territorial Hospital Center of Magenta, Noumea, New Caledonia

## Background

Worldwide, lower respiratory infections (LRIs) are the most frequent cause of death in children under 5 years. The Pneumonia Etiology Research for Child Health (PERCH) project is a large, multi-center case-control study of hospitalized pediatric patients with severe LRI to determine the etiology and risk factors associated with the syndrome. By applying modern tools with standardized methods, PERCH will contribute to new, precise information to guide the development of future vaccines and treatments. A pilot study aims to describe LRI etiologies in New Caledonia and to evaluate new diagnostic techniques.

## Methods

We started a 1-year case-control study in children aged 1 month to 15 years. We collected induced sputum (IS) with forced expiratory flow, nasopharyngeal aspiration (NPA), urine and blood from cases hospitalized with pneumonia or bronchiolitis, and NPA and blood from controls without respiratory infection. Bacteriological tests consist of blood and respiratory specimen culture and urinary antigen detection for *L. pneumophila*. NPA and IS were tested for *B. pertussis*, *Mycoplasma pneumoniae* and *Chlamydophila pneumoniae* using PCR. Virus detection was performed using fluorescence and PCR (multiplex and mass-tag). Antibody detection (for *M.*/*C. pneumoniae* and respiratory viruses) was performed on acute and 30-day convalescent sera.

## Results

Within 6 months, 19 controls and 80 cases (56 bronchiolitis and 24 pneumonias) were enrolled. The median age was 9 months [range 1 month – 11 years]. At least one respiratory pathogen was found in 81% of cases and 33% of controls. Bacterial pathogens were found in 50% of cases with pneumonia. S.pneumoniae and H.influenzae were the most frequently found bacteria. Viruses were identified in 29% of cases among which 50% were RSV. Viral/bacterial co-infections occurred in 8% of cases. Among children with bronchiolitis, RSV was the most frequently found virus (90%) with an epidemic peak in April-May, the beginning of the cooler season.

## Conclusion

Diagnostic testing methods enabled detection of possible etiology in most of the ARI case.

